# Silyldienolates in Organocatalytic Enantioselective Vinylogous Mukaiyama-Type Reactions: A Review

**DOI:** 10.3390/molecules26226902

**Published:** 2021-11-16

**Authors:** Leon Hoppmann, Olga García Mancheño

**Affiliations:** Organic Chemistry Institute, University of Münster, Corrensstraße 36, 48149 Münster, Germany; leon.hoppmann@uni-muenster.de

**Keywords:** silyldienolates, vinylogous reactions, organocatalysis, asymmetric catalysis

## Abstract

Mukaiyama aldol, Mannich, and Michael reactions are arguably amongst the most important C–C bond formation processes and enable access to highly relevant building blocks of various natural products. Their vinylogous extensions display equally high potential in the formation of important key intermediates featuring even higher functionalization possibilities through an additional conjugated C–C double bond. Hence, it is much desired to develop highly selective vinylogous methods in order to enable unconventional, more efficient asymmetric syntheses of biologically active compounds. In this regard, silyl-dienolates were discovered to display high regioselectivities due to their tendency toward *γ*-additions. The control of the enantio- and diastereoinduction of these processes have been for a long time dominated by transition metal catalysis, but it received serious competition by the application of organocatalytic approaches since the beginning of this century. In this review, the organocatalytic applications of silyl-dienolates in asymmetric vinylogous C–C bond formations are summarized, focusing on their scope, characteristics, and limitations.

## 1. Introduction

**Scheme 1:**
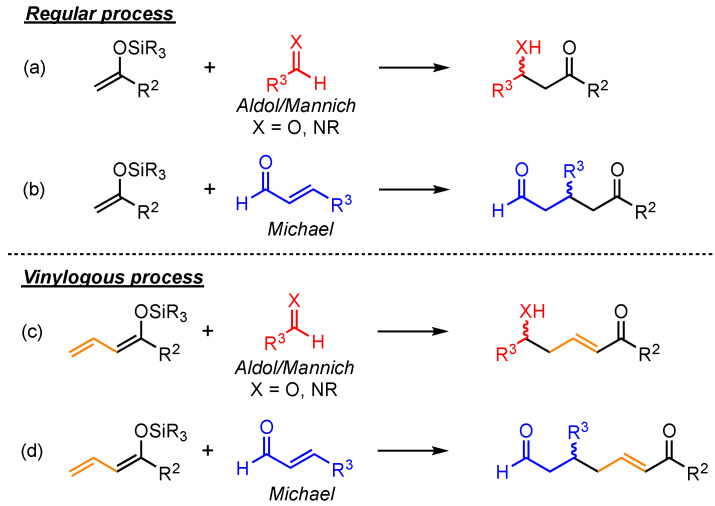
Vinylogous extension of common Mukaiyama aldol, Mannich, and Michael C–C bond formation reactions.

The principle of vinylogy, demonstrated by Fuson in 1935, explains that the integration of conjugated C=C-double bonds next to functional groups allows moving their intrinsic reactive site to a more distant point within the molecule [1]. Applying this concept to basic reaction types enables various new pathways to relevant structural motifs. Arguably, the most explored vinylogous versions are reported for relatively simple C–C bond formations, especially for aldol, Mannich, and Michael reactions [2,3,4,5,6]. In contrast to the usual observed *α*-additions to the respective electrophiles in these reactions, the vinylogous extension gives rise to the competing, often favored *γ*-additions (Scheme 1). Hence, due to elongated carbon chains and the assured presence of *α*,*β*-unsaturated carbonyl-moieties, this method allows for the formation of more complex and versatile products.

The application of vinylogy to regular C–C bond formations has provided important and synthetically less tedious pathways to structural motifs that commonly occur in natural products. Among these, one of the most commonly investigated moieties is the butenolide, which is formed by the *γ*-addition of furan-based dienolates to the corresponding electrophiles. These *γ*-butenolides represent important subunits in various natural compounds with essential biological activities (e.g., avenolides (antibiotic), kalloides (anti-inflammatory) or Arglabins (anti-tumor)) [7,8,9,10,11]. The application of acyclic dienolates is of similar significance, as it gives rise to analog linear functionalities. Hence, the employment in vinylogous aldol reactions gives access to extended polyol- or polyketide-subunits, furnishing elegant alternatives to common enzymatic approaches [6]. Vinylogous Mannich reactions instead provide the simple formation of *δ*-amino-*β*-ketoesters, which are used as building blocks in the synthesis of different alkaloids [12]. Finally, the utilization of acyclic dienolates in vinylogous Michael reactions allows for the formation of 1,7-dioxo-compounds, which also feature valuable key intermediates in pathways toward natural or medicinal products [13].

**Figure 1:**
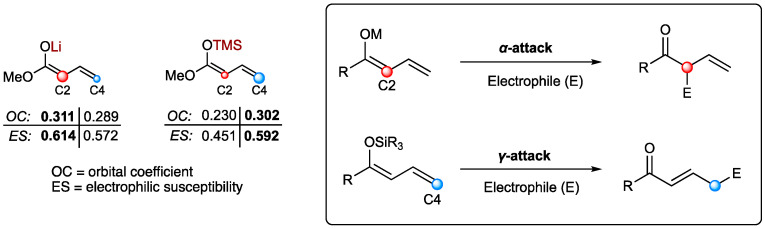
Intrinsic reactivity of different types of dienolates.

To fulfill their purpose of providing building blocks and intermediates for the synthesis of natural compounds, highly selective methods are required. As stated earlier, the vinylogous extension enables an additional reaction site, thus giving rise to two different regioisomers. Remarkably, investigating the nature of the employed dienolates led to the discovery of differences in regioselectivity [14]. Hence, metal-based dienolates favor *α*-additions, while silyl-protected nucleophiles tend to favor *γ*-additions (Figure 1). This phenomenon can further be underlined by computational calculations of the frontier molecular orbital density [15]. Since the corresponding *γ*-addition products are often more desirable than their *α*-analogs, the silyl-dienolates emerged as the superior nucleophile species in vinylogous reactions.

Another essential aspect is the ability to control the diastereo- and enantioselectivity within the addition products. Since most of the concerned reactions already proceed better in the presence of reactivity-enhancing catalysts, this principle was expanded to asymmetric catalysis in order to obtain the desired chiral products. Although first approaches in this matter exclusively featured the employment of metalorganic catalysts, asymmetric vinylogous C–C bond formation reactions were progressively taken over by organocatalytic applications since the beginning of this century [16,17,18,19,20].

This review summarizes the development of asymmetric organocatalyzed C–C bond formations, namely Mukaiyama aldol, Mannich, and Michael reactions, focusing on the addition of both cyclic and acyclic silyl-dienolates. The related organocatalytic (hetero)-Diels–Alder reactions with silyl-dienolates have previously been extensively reviewed and therefore are not included here [21,22,23].

In contrast to earlier overviews in this field, this work presents the first review that exclusively focuses on organocatalytic applications and the employment of silyl-protected dienolate nucleophiles. Thus, a specific outline is provided, reflecting the detailed development of the featured methods since their pioneering work.

## 2. Vinylogous Mukaiyama Aldol Reactions

The earlier mentioned motifs of polyols and polyketides represent important building blocks due to their biological activity. In nature, these functions are formed by perfectly adjusted enzymatic processes, providing high efficiency and selectivity. According to this, the challenge for the non-enzymatic synthetic formation of these motifs arises, as comparable product purity is required. The aldol reaction was found to be one of the most suitable methods for this requirement, since it allows the highly selective formation of 1,3-diol relationships and thus supports the synthesis of the desired polyol/polyketide-structures. The extension to vinylogous aldol reactions further expands the versatility of the obtained products. It provides *δ*-hydroxy-*β*-ketoesters and *δ*-hydroxy-*α*,*β*-unsaturated carbonyls, which further enable desirable derivatization through various follow-up reactions. Nevertheless, the challenging induction of high regio-, diastereo-, and enantioselectivities to aldol reactions still features wide interest, especially embracing catalytic approaches [24].

**Scheme 2:**
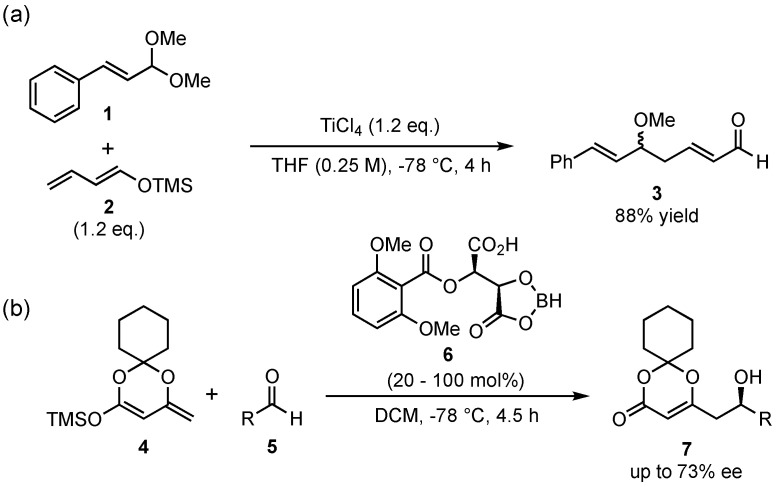
The original vinylogous Mukaiyama aldol reaction (VMAR) presented by Mukaiyama and Ishida (**a**) [25] and its first enantioselective version by Kaneko and coworkers (**b**) [28].

The first vinylogous Mukaiyama aldol reaction (VMAR) was published in 1975 by Mukaiyama and Ishida, who presented the addition of the crotonaldehyde-based silyl-dienolate **2** to cinnamaldehyde dimethyl acetal (**1**) in the presence of super-stoichiometric amounts of TiCl_4_ (Scheme 2a) [25]. This finding was the beginning of a very promising reaction type in organic chemistry and has since then found many applications in important syntheses [16,17,18,19,24,26,27]. Although the first examples featured only racemic reactions, the potential for asymmetric applications was already at hand, given the tunable catalytic reaction mechanism. Thus, the first enantioselective approach was published by Kaneko et al. in 1994, in which they presented the reaction between the cyclic silyl-dienolate **4** and different aldehydes **5**, which were catalyzed by chiral borane complexes **6** (Scheme 2b) [28].

**Scheme 3:**
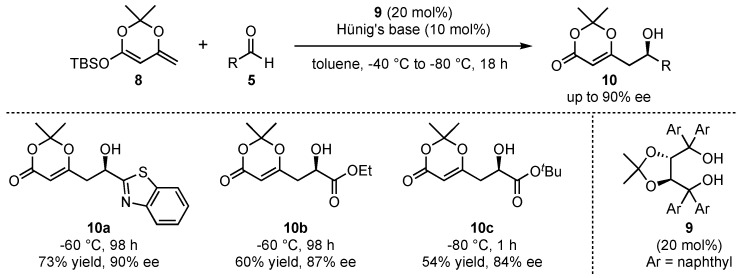
First asymmetric organocatalyzed VMAR pioneered by Rawal et al. [29].

More than 10 years later, when the field of organocatalysis had found broad recognition in synthetic organic chemistry, Rawal et al. pioneered the organocatalyzed application of silyl-dienolates in asymmetric VMARs (Scheme 3) [29]. They utilized similar cyclic dienolates **8** as in the earlier work of Kaneko et al. and subsequently investigated its reaction with various aldehydes **5**. In this regard, chiral alkaloid- or diol-based organocatalysts were utilized to induce high enantioselectivities. It was found that the best results were obtained with α,α,α’,α’-tetraaryl-2,2-disubstituted 1,3-dioxolane-4,5-dimethanols (TADDOLs) **9** at low temperatures (−60 to −80 °C), which resulted in the products **10** in up to 90% ee under otherwise optimized conditions. Interestingly, the reaction with glyoxalates (products **10b** and **10c**) was assisted by the addition of Hünig’s base (*N*,*N*-diisopropylethylamine) in order to slow down the racemic background reaction by trapping water or other acidic compounds being formed during the process. A further advantage of the reaction is featured by its excellent regioselectivity, since the formed products exclusively show *γ*-addition of the nucleophiles.

**Scheme 4:**
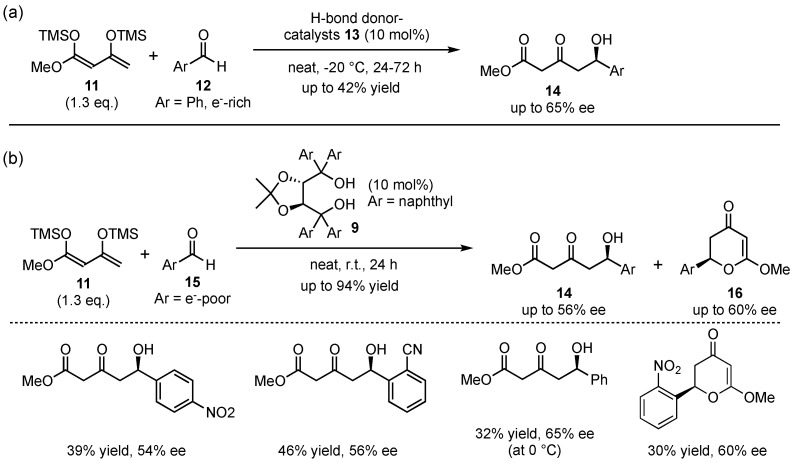
Asymmetric organocatalyzed VMAR reaction between Chan’s diene and electron-rich and poor aldehydes by Scettri et al. [30,33].

Employing the same catalyst type, the group of Scettri investigated the reaction behavior of Chan’s diene (**11**) in an organocatalyzed asymmetric VMAR (Scheme 4) [30]. Although this group earlier utilized a chiral phosphoramide/SiCl_4_ catalyst system [31,32], in 2009, they introduced this particular dienolate to several electron-rich and electro-neutral benzaldehydes **12** in the presence of different H-bond-donor catalysts **13** and observed the exclusive formation of the aldol product **14** in moderate yields and ees (Scheme 4a). Interestingly, the reaction with electron-poor benzaldehydes **15** yielded a mixture of the expected aldol-product **14** and a cyclized dihydro-pyrone **16** in overall good yields and moderate enantioselectivities up to 56% ee and 60% ee, respectively (Scheme 4b). The formation of the unexpected side product was traced back to a hetero-Diels–Alder reaction (HDA), giving rise to the formation of an alternative pyrone regio-isomer compared to the one usually obtained by the reaction of Brassard’s diene with aldehydes [33].

**Scheme 5:**
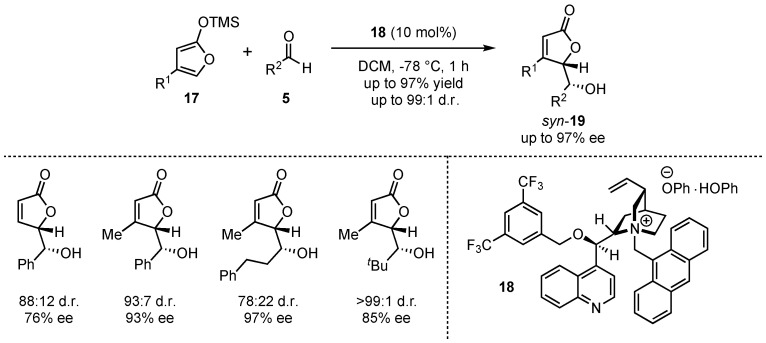
Lewis-base organocatalyzed asymmetric VMAR by Mukaiyama et al. [34].

In 2007, Mukaiyama et al. presented the first employment of a chiral Lewis base organocatalyst **18** in a VMAR (Scheme 5) [34]. Their research showed that the reaction of 4-substituted 2-(trimethylsiloxy)furans **17** with different aldehydes **5** at low temperatures (−78 °C) provided good yields (up to 97%) and enantioselectivities (up to 97% ee), while the diastereoselectivity was moderate with just a few exceptions.

**Scheme 6:**
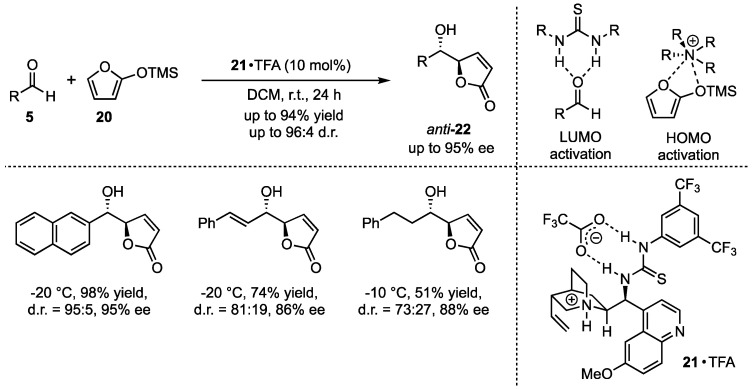
Cinchona alkaloid carboxylate organocatalyzed asymmetric VMAR presented by Deng et al. [35].

Subsequently, the group of Deng addressed open challenges of this method, namely the limited investigation of functional group tolerance concerning both substrates and a missing anti-selective procedure of this reaction. In this regard, they applied bifunctional cinchona alkaloid-based catalysts **21** in the presence of carboxylic acids (Scheme 6) [35]. They proposed an activation mechanism, in which the silyl dienolate is activated by the protonated quinuclidine, while the thiourea moiety activates the aldehyde by H-bond lowest unoccupied molecular orbital (LUMO) lowering. By employing this optimized new catalyst, they obtained excellent yields and selectivities in the reactions between 2-(trimethylsilyloxy)furan (**20**) and different aldehydes **5**. It is worth mentioning that both aromatic and aliphatic substitutions were well tolerated.

**Scheme 7:**
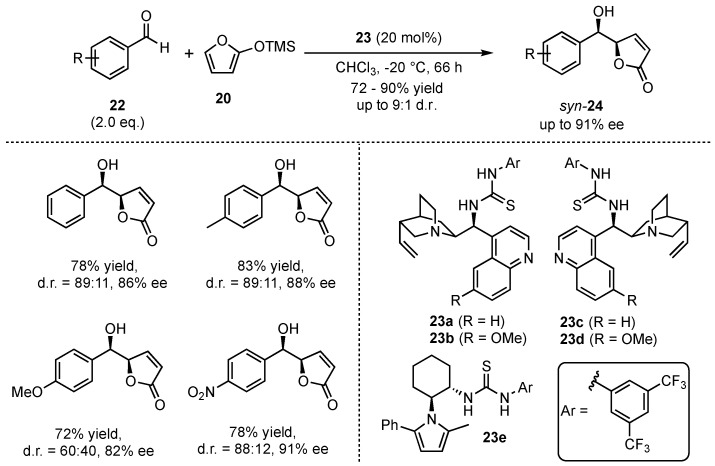
Asymmetric VMAR between 2-(trimethylsilyloxy)furan and aromatic aldehydes organocatalyzed by bifunctional thioureas by Wang et al. [36].

At the same time, Wang et al. investigated the reaction of 2-(trimethylsilyloxy)furan (**20**) with several aromatic aldehydes **22** in the presence of neutral bifunctional thiourea organocatalyst **23a**–**e** (Scheme 7) [36]. Under optimized reaction conditions, they were able to achieve high yields (72–90%), diastereomeric ratios (up to 9:1 toward the *syn*-product) and enantioselectivities (82–91% ee) in the corresponding aldol products **24**. The reaction proceeded well either with electron-rich and electron-poor aldehydes, displaying a broad scope and functional group tolerance.

**Scheme 8:**
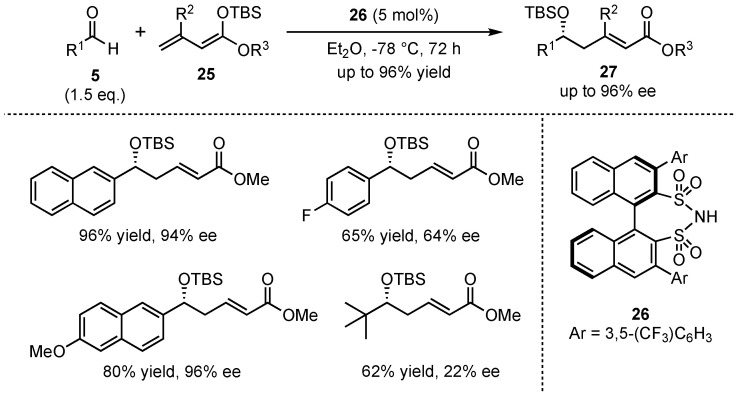
Enantioselective VMAR catalyzed by a disulfonimide organocatalyst, presented by List et al. [37].

In 2011, List et al. presented a VMAR between aromatic aldehydes **5** and acyclic silyl-dienolates **25** in the presence of their earlier developed disulfonimide organocatalyst **26** (Scheme 8) [37]. It was discovered that neither the *E*/*Z*-composition nor the silyl-protecting group of the dienolate had a large influence on the reaction. In contrast, ester moieties with higher steric demand (e.g., *i*Pr or *t*Bu) and substitution in the *β*-position lowered the yield significantly, while substitution in the *α*-position gave inferior enantioselectivity. Furthermore, electro-neutral and electron-rich aromatic aldehydes were more suitable than aliphatic substrates in terms of both yield (up to 96%) and enantioselectivity outcome (up to 96% ee). It is worth mentioning that most of the received aldol products **27** showed excellent *α*/*γ*-ratios of up to >40:1.

**Scheme 9:**
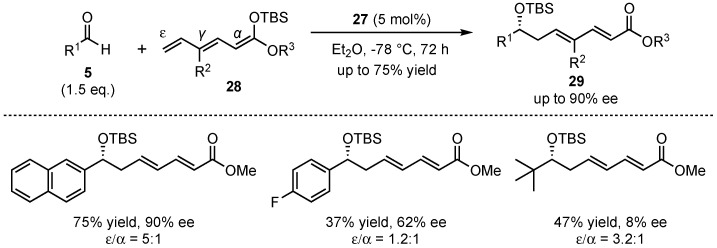
First organocatalyzed enantioselective bis-vinylogous Mukaiyama aldol reaction.

The group was able to expand this method to a bis-vinylogous Mukaiyama aldol reaction, featuring silyl-protected trienolates **28** and different aldehydes **5** (Scheme 9). In contrast to the earlier presented study, the obtained regioselectivities (mixture of ε- and *α*-adducts) were only moderate. Nevertheless, good yields (up to 75%) and excellent enantioselectivities (up to 90% ee) in the aldol-products **29** were achieved for a broad range of substrates. It was shown that again, electro-neutral and electron-rich aromatic aldehydes provided the best results, while electron-deficient and especially aliphatic substrates suffered from bad yields and enantioselectivities. In general, it was examined that this bis-vinylogous Mukaiyama aldol reaction yielded inferior results than its regular vinylogous version under the same conditions, but it still bears potential for future investigations.

**Scheme 10:**
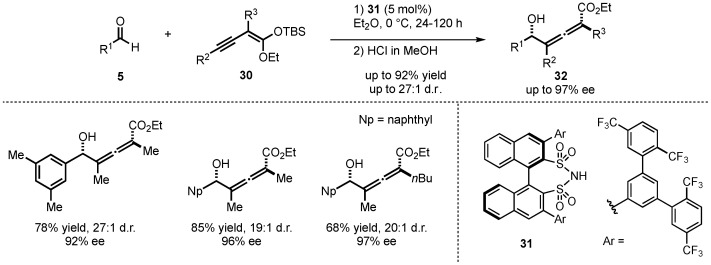
First enantioselective alkynylogous Mukaiyama aldol reaction organocatalyzed by disulfonimides presented by List et al. [38].

Furthermore, List et al. also investigated the closely related alkynylogous Mukaiyama aldol reaction between silyl alkynyl ketene acetals **30** and different aldehydes **5** (Scheme 10) [38]. They employed the earlier displayed disulfonimide catalyst class **31** to generate tetra-substituted allenes **32** with excellent yields (up to 92%), diastereoselectivities (up to 27:1), and enantioselectivities (up to 97% ee).

**Scheme 11:**
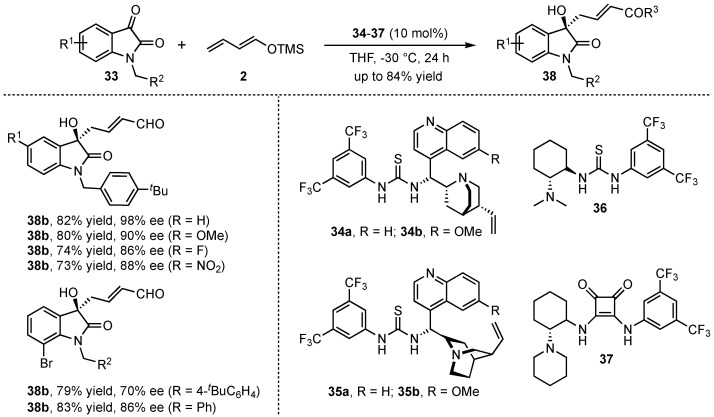
Enantioselective VMAR of isatins by bifunctional H-bond donor organocatalysis presented by Alemán et al. [39].

More recently, the group of Alemán investigated the organocatalyzed VMAR of isatins **33** with the linear crotonaldehyde derived dienolate **2** in the presence of chiral bifunctional thioureas and squaramides (**34**–**37**) (Scheme 11) [39]. After detailed optimization studies featuring different catalysts, solvents, isatin-*N*-substitutions, and the addition of H_2_O, the corresponding aldol products **38** were obtained in good yields (up to 82%) and excellent enantioselectivities (up to 98% ee). Then, a broad scope of different substitutions in the aromatic moiety revealed that both electron-donating and electron-withdrawing groups were well tolerated. The only exception was found for steric hindered 7-substituted substrates that disturb an efficient coordination of the catalyst, leading to diminished enantioselectivities.

## 3. Vinylogous Mukaiyama Mannich Reactions

The Mannich reaction is closely related to the aldol reaction and mainly deviates in the nature of the applied electrophile, either featuring imines or iminium ions. Ergo, the reaction products later exhibit primary or secondary amine moieties. The regular Mannich reaction leads to the formation of biologically relevant *β*-amino-carbonyl compounds (e.g., *β*-amino-acids). A vinylogous extension of the reaction further enables the synthesis of *δ*-amino-*α*,*β*-unsaturated carbonyl compounds, which represent highly functionalized intermediates in the preparation of various natural compounds (e.g., alkaloids). Vinylogous Mannich processes are not explored as detailed as the related aldol reactions, yet their versatility and synthetic significance raise high interest especially in search of stereoselective catalytic applications [5].

**Scheme 12:**
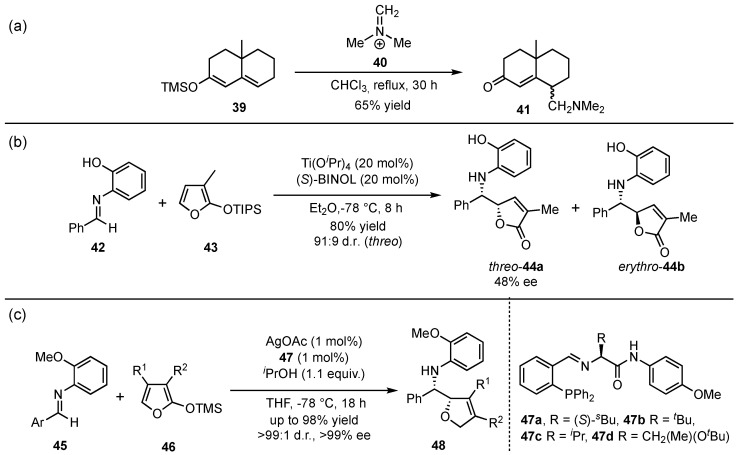
The origin of the vinylogous Mukaiyama Mannich reaction (VMMnR) by Danishefsky et al. (**a**) [40], pioneering of an asymmetric approach by Martin et al. (**b**) [41], and the first highly enantioselective application presented by Hoveyda and Snapper (**c**) [42].

In 1980, the group of Danishefsky presented the first vinylogous Mannich reaction between silyl-protected dienolates **39** and Eschenmoser iminium salt (**40**), delivering the corresponding *γ*-methylenketones **41** in yields up to 65% (Scheme 12a) [40]. First in 1999, Martin et al. reported pioneering work in the field of asymmetric vinylogous Mukaiyama Mannich reactions (VMMnR) by applying chiral organometallic catalysis (Scheme 12b) [41]. Under optimized conditions for the reaction between an aldehyde-based aromatic imine **42** and triisopropylsilyloxyfuran **43**, the threo (**44a**) and erythro adducts (**44b**) were obtained in a ratio of 91:9 and 48% ee within the major product (**44a**). Since only moderate enantioselectivities were accomplished in this first approach, the search for a highly selective method was further pursued. In this regard, seminal investigations were presented by Hoveyda and Snapper in 2006, in which AgOAc was used as a Lewis acid catalyst in the presence of chiral phosphine ligands **47** [42]. They applied their design on a similar reaction and, in contrast, managed to raise the yields, diastereo- and enantioselectivities of the corresponding *γ*-butenolides **48** (Scheme 12c).

In the following years, the asymmetric VMMnR was investigated by many different work groups [5,19,43,44]. However, most of the publications featured organometallic catalysis or the asymmetric induction by chiral auxiliars. Thus, only a limited number of organocatalytic applications in asymmetric VMMnRs with silyl-protected dienolates have been published to date.

**Scheme 13:**
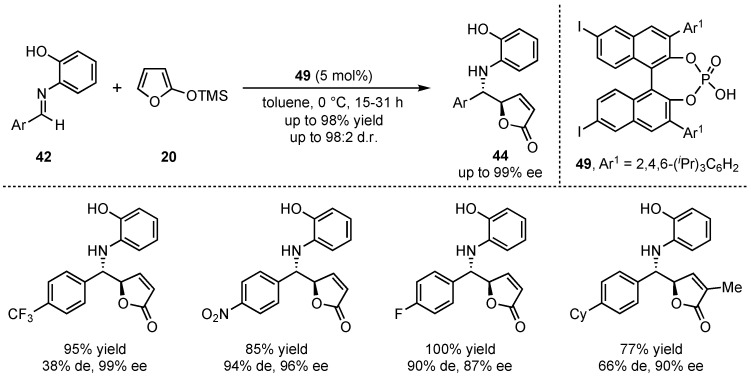
First organocatalytic approach of the asymmetric VMMnR in the formation of *γ*-butenolides by Akiyama et al. [45].

In this regard, the group of Akiyama presented a novel organocatalyzed asymmetric formation of *γ*-butenolides **44** via a VMMnR [45]. In detail, they applied an iodine substituted chiral phosphoric acid **50** to the reaction between 2-(trimethylsilyloxy)furan (**20**) and different aldimines **42** (Scheme 13). While an *ortho*-hydroxy group was required at the *N*-aryl imine for attaining high yields, diastereo- and enantioselectivities, electron-poor aromatic aldimines granted better results than electro-neutral aromatic and aliphatic substrates.

**Scheme 14:**
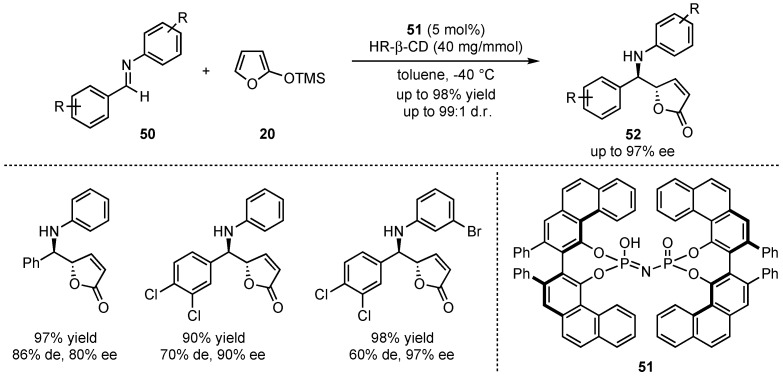
Improved method for the organocatalyzed asymmetric VMMnR between aromatic aldimines and silyl-dinolates renouncing the necessity of neighboring hydroxy-groups presented by Zhang et al. [46].

Later on, the group of Zhang urged for a method that does not rely on the mandatory presence of a neighboring hydroxy group at the imine for dual hydrogen-bonding interactions [46]. In this regard, promising results were obtained by applying imidophosphoric acid catalysts, which were earlier pioneered by List et al. [47]. More specifically, the 2,2′-diphenyl-3,3′-biphenanthryl-4,4′-diylphosphate (VAPOL)-derived Brønsted acid catalyst **51** induced the best results in asymmetric VMMnR’s between 2-(trimethylsilyloxy)furan (**20**) and several different aromatic aldimines **50** (Scheme 14). The demonstrated broad scope showed a high tolerance for substitutions and functional groups, furnishing the products in high yields (up to 98%), diastereo- (up to 99:1 d.r.) and enantioselectivities (up to 97% ee). Especially, a 3,4-dichloro substitution in the aldimine core led to excellent results within the respective *γ*-butenolides **52**.

**Scheme 15:**
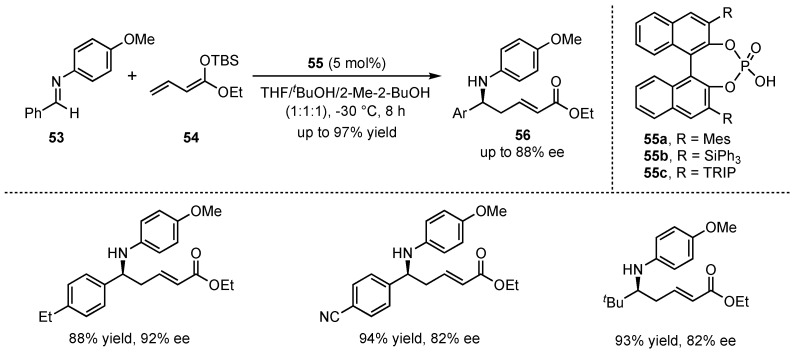
Brønsted acid organocatalyzed asymmetric VMMnR’s between aldimines and linear silyl-dienolates by Schneider et al. [48].

Simultaneously to the earlier illustrated work of Akiyama et al., the group of Schneider published their investigations on the organocatalytic application of chiral phosphoric Brønsted acids **55** in a VMMnR. However, they exhibited the first utilization of linear silyl-dienolates **54** in this field [48]. After detailed optimization studies on the reaction conditions, they exclusively received the corresponding *δ*-amino *α*,*β*-unsaturated carboxylic acid derivatives **56** in high yields (up to 97%) and enantioselectivities (up to 92% ee) (Scheme 15). It was discovered that polar-protic solvents (e.g., *t*BuOH) were mandatory for the catalytic activity of the applied Brønsted acids. Furthermore, different substitutions on the employed aldimines **53** did not diminish the results, thereby demonstrating the broad applicability of this method. In a later publication, this reaction was utilized as a prototype for the development of the first asymmetric organocatalytic reaction on a single microfluidic nanospray chip with integrated enantiomer separation and nanoES-MS-analysis [49].

Between 2008 and 2011, the group of Schneider further extended their investigations on this type of reaction toward higher enantioselectivities and broader scope on both the aldimine substrates and dienolate nucleophiles [50,51,52]. Additionally, it was discovered that the reaction can also be carried out in a three-component fashion, thereby avoiding the use of preformed imines and saving one synthesis step [51].

**Scheme 16:**
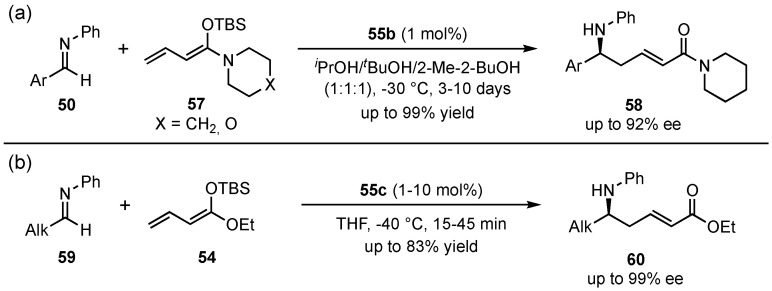
Investigation of linear vinylketene silyl *N*,*O*-acetals in Brønsted acid organocatalyzed asymmetric VMMnRs (**a**) and optimization in the utility of aliphatic substrates (**b**) by Schneider et al. [50,52].

Next to the usually applied ester-derived dienolates, this group also investigated the employment of other dienolate types [50]. Especially, piperidine and morpholine-based vinylketene silyl *N*,*O*-acetals **57** proved to be good alternatives and provided excellent yields (up to 99%) and enantioselectivities (up to 92% ee) with an exclusive *γ*-selectivity (Scheme 16a). Furthermore, they also encountered the issue of poor results with aliphatic aldimines [52]. In this regard, the solvent mixture was changed to THF, since protic solvents promote enolization of the aldimines and subsequent self-aldolization, explaining the overall poor yields. A following optimization further revealed that bulkier catalysts **55c** in the presence of ester-based dienolates **54** furnished the best enantioselectivities (91% ee) (Scheme 16b). Then, with this improved method, aliphatic substrates **59** showed overall good yields (up to 83%) and excellent enantioselectivities (up to 99% ee). Interestingly, aromatic aldimines **50** featured no reaction at all under these conditions.

**Scheme 17:**
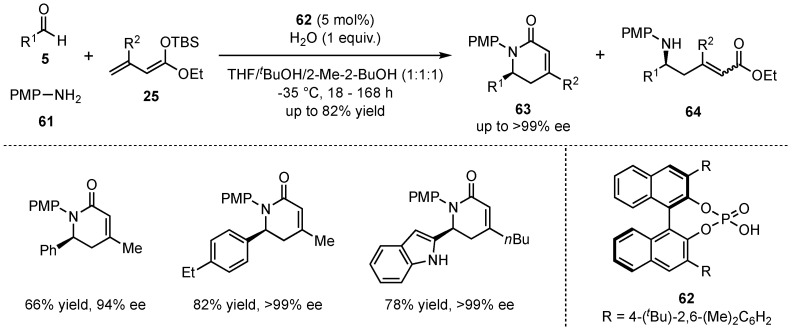
Formation of aza-Diels–Alder cycloadducts by alternative reaction control in the presence of chiral phosphoric Brønsted acids.

In 2017, Schneider et al. discovered that *β*-alkylated dienolates **25** also furnish aza-Diels–Alder adducts in the earlier mentioned three-component VMMnRs under otherwise unchanged conditions [53]. In a catalyst screening, it was discovered that by employing chiral phosphoric Brønsted acid **62**, the reaction can be controlled to primarily form the desired *N*-heterocycles **63**. Hence, the cyclic products were obtained in high yields (up to 82%) and excellent enantioselectivities (up to >99:1) (Scheme 17).

**Figure 2:**
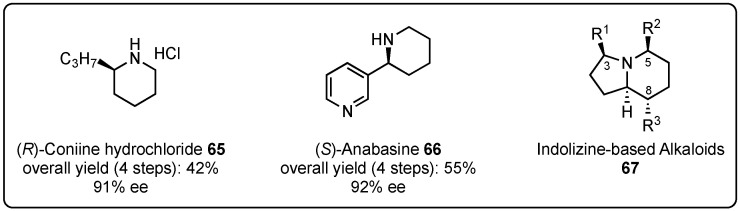
Total synthesis of (*R*)-coniine hydrochloride (**65**), (*S*)-anabasine (**66**), and indolizine-based alkaloids (**67**) based on initial Brønsted acid organocatalyzed asymmetric VMMnRs by Schneider et al. [52,54,55].

In order to demonstrate the synthetic relevance of this reaction, the Schneider group embraced their method for the synthesis of known natural compounds that commonly require more complex or additional reaction steps. In this regard, they accomplished the synthesis of (*R*)-coniine hydrochloride (**65**) [52] and (*S*)-anabasine (**66**) [54] in moderate yields and excellent enantioselectivities (Figure 2). Furthermore, they intensively studied the synthetic access to indolizine-based alkaloids (IBAs) **67** with several different substitution patterns, mainly in the 3-, 5-, and 8-position [55]. A VMMnR is the key step of this synthesis route and was optimized in the presence of chiral 1,1′-bi-2-naphthol (BINOL)-based phosphoric acids to provide the desired lactam intermediates in good yields and excellent diastereo- and enantioselectivities (up to >99% ee). Building on these enantiopure intermediates, the further incorporation of substituents and chiral centers was achieved under substrate control, which led to the formation of several natural-occurring alkaloids with high purity.

**Scheme 18:**
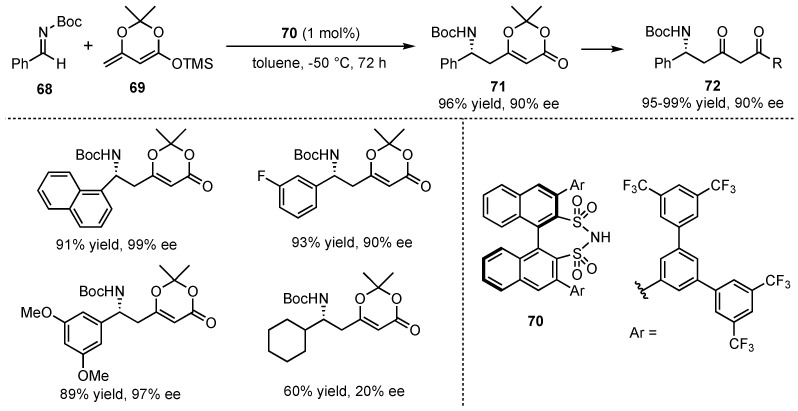
Chiral disulfonimide organocatalyzed VMMnRs and subsequent transformation to useful enantiomerically pure building blocks presented by List et al. [12].

In 2014, the group of List presented an asymmetric VMMnR catalyzed by their developed Brønsted acid disulfonimide catalyst **70**, which was already successfully used in an earlier work on VMARs (Scheme 7) [12]. This time, the catalyst was applied to the reaction between the cyclic dienolate **69** and different *N*-Boc imines **68** (Scheme 18). After a detailed catalyst and broad substrate screening, the corresponding *δ*-amino-*α*,*β*-unsaturated cyclic ester products **71** were obtained in excellent yields and enantioselectivities (both up to 99%) with just a small exception regarding aliphatic substrates. In follow-up studies, further reactions on the received products provided the corresponding dioxinones **72** with maintained high enantiopurity.

Later on, the group applied the same conditions to the reaction between the acyclic Chan’s diene (**11**) and similar aromatic *N*-Boc-aldimines [56]. The following substrate scope underlined the versatility of the reaction, since the application of different dienolates still provided excellent yields and enantioselectivities with over 90% ee.

**Scheme 19:**
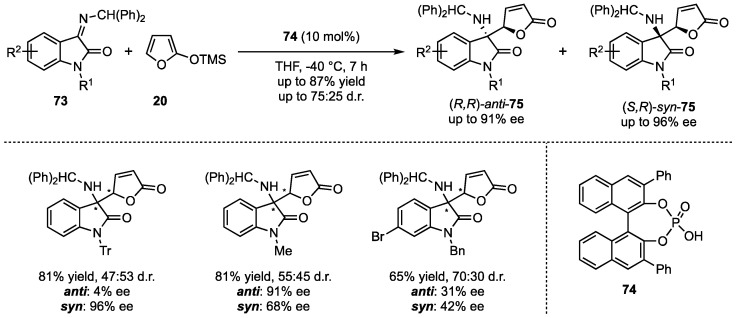
First organocatalyzed asymmetric VMMnR on ketimine-based substrates, presented by Silvani et al. [57].

In contrast to the earlier featured reactions with exclusively employed aldimine-substrates, Silvani et al. presented the first enantioselective VMMnR with less reactive, more challenging ketimines [57]. More specifically, additions of 2-(trimethylsiloxy)furan (**20**) to isatine-derived benzhydryl isatins **73** were carried out in the presence of chiral phosphoric acids **74** (Scheme 19). The stereocontrol of this reaction bears higher complexity due to the larger steric demand and lower reactivities compared to aldimines substrates. Interestingly, the stereocontrol was regulated by the reaction temperature. At high temperatures, racemic mixtures with excellent d.r. were obtained, while for lower temperatures (down to −78 °C), this tendency was reversed. A subsequent substrate screening revealed that isatin-*N*-substitutions were very well tolerated regarding the yield (up to 81%) and the enantioselectivity (up to 96% ee), while modifications in the aromatic core only led to the products **75** in moderate yields (42–68%) and decreased enantioselectivities (1–74% ee).

**Scheme 20:**
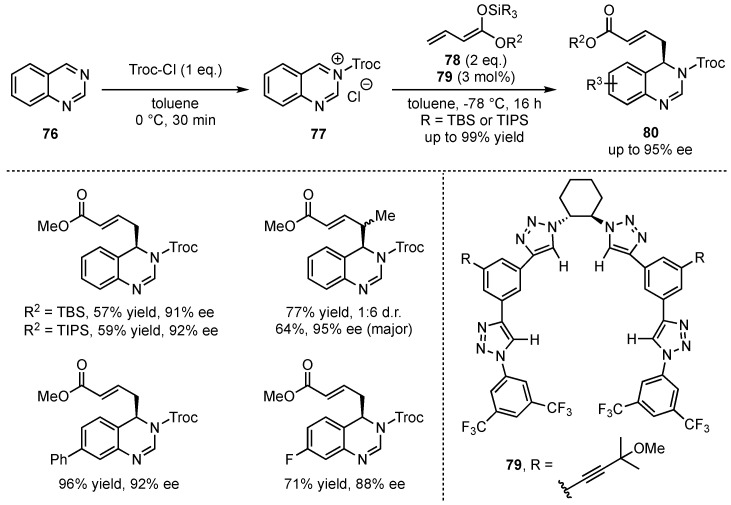
Asymmetric anion-binding catalyzed vinylogous Mukaiyama Mannich-type dearomatization by Alemán and García Mancheño [58].

Only recently, the groups of Alemán and García Mancheño presented the dearomatization of quinazolines in an asymmetric vinylogous Mukaiyama Mannich-type reaction following a Reissert-type approach by first acylation of the substrates **76** and subsequent nucleophilic addition of silyl-dienolates **78** to the in situ formed iminium ion **77** (Scheme 20) [58]. The reactions were performed in the presence of chiral tetrakistriazole-based anion-binding catalysts **79** giving the vinylogous products **80** in excellent yields (up to 99%) and enantioselectivities (up to 95% ee). After comprehensive optimization studies, the reaction was carried out with different silyl-dienolates. Especially, bulky ester moieties and silyl-protecting groups yielded good results, while *α*-, *β*-, and *γ*-methylation was also well tolerated. Eventually, the relevance of this new method was further broadened by successful upscaling, derivatization, and catalyst-recycling reactions.

## 4. Vinylogous Mukaiyama Michael Reactions

In contrast to the earlier displayed Mukaiyama aldol (VMAR) and Mannich (VMMnR), the equivalent vinylogous Michael reaction (VMMcR) is not explored in such a broad fashion, which is mostly caused by the more difficult control of its stereochemical outcome. Since both substrates feature two different reaction sites (*α*- or *γ*-reactivity for the dienolate nucleophile and 1,2- or 1,4-reactivity regarding the carbonyl electrophile), these reactions potentially result in four different regioisomers. Although this obstacle is challenging to overcome, there is still a high potential within this method, as it enables the synthesis of various chiral 1,7-dioxo compounds. Thus, this topic has found large interest in the chemical community for the last 20 years [4,18,59].

**Scheme 21:**
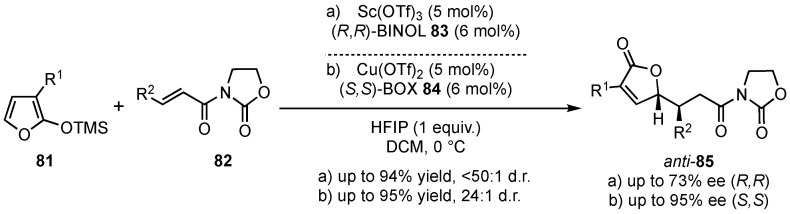
First enantioselective vinylogous Michael reaction (VMMcR) presented by Katsuki et al. [60].

The first enantioselective approach toward the VMMcR was already proposed in 1997 by Katsuki et al., in which they presented the Lewis-acid catalyzed addition of 2-(trimethylsilyloxy)furans **81** to oxazolidone enoates **82** (Scheme 21) [60]. A detailed screening, primarily catalyzed by scandium triflates in the presence of different BINOL-derived ligands **83**, yielded the sought-after enantioinduction but failed to go above moderate selectivities (up to 73% ee). However, with this method, the desired *γ*-butenolides **85** were received in excellent diastereomeric ratios (>50:1). Later on, the enantioselectivity of the process could be improved to give the respective products with up to 95% ee by switching the catalyst system to copper triflate in combination with chiral bis(oxazoline)-ligands **84**, although the diastereomeric ratios were diminished.

**Scheme 22:**
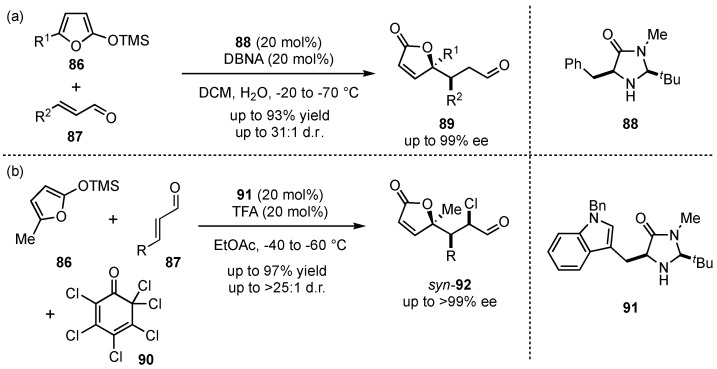
First highly enantioselective organocatalyzed VMMcR (**a**) and subsequent one-pot chlorination through cascade reactions by MacMillan et al. (**b**) [63,64].

Apart from this example by Katsuki, most metal-based Lewis acid-catalyzed VMMcR’s tend to facilitate 1,2- rather than 1,4-additions to unsaturated carbonyls [61,62]. In order to overcome this issue, the group of MacMillan reported in 2003 a vinylogous Michael method relying for the first time on organocatalysis [63]. Therein, they proved that the employment of secondary amine catalysts **88** and the in situ formation of aldehyde-based iminium ions facilitate the corresponding 1,4-addition (Scheme 22a). With the desired selectivity in hand, the flexibility of this process was explored. It was shown that acylated or alkylated siloxy-furans in 5-position **86**, as well as *γ*-substituted aldehydes **87**, provided **89** in high yields (up to 93%) and excellent enantioselectivities (up to 99% ee) while maintaining good diastereoselectivities (up to 31:1 d.r.). Ultimately, they demonstrated the importance of this method by applying it to the synthesis of the commercially relevant spiculisporic acid.

Later on, the same group developed a cascade-VMMcR, in which iminium-ion and enamine catalysis were merged by exclusively applying one imidazolidone-catalyst **91** [64]. Thereby, the asymmetric addition of 5-methyl-2-(trimethylsilyloxy)furan (**86**) to different aldehydes **87** and subsequent chlorination in the *α*-position through chlorinated quinone **92** was achieved in a one-pot reaction (Scheme 22b). The corresponding products **92** were obtained in high yields (up to 97%) and with exceptional enantiomeric excesses (>99% ee).

**Scheme 23:**
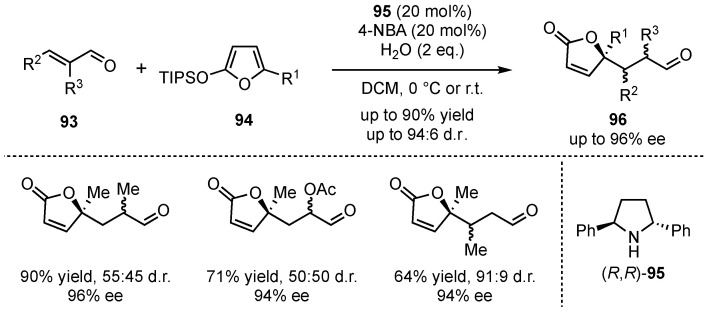
Extension of the secondary amine-catalyzed VMMcR to *α*-substituted enal-substrates by Pápai and Pihko [65].

Almost 10 years later, a series of related articles were published [65,66,67]. The groups of Pápai and Pihko focused on the challenging stereocontrol of a VMMcR with *α*-substituted enals [65]. After intensive studies, *trans*-2,5-diphenylpyrrolidine (**95**) was found to be a suitable catalyst for the reaction between TIPS-protected silyloxyfurans **94** and *α*-substituted enals **93** (Scheme 23). It was discovered that 10–20 mol% of catalyst was sufficient to provide the products **96** in good yields (up to 90%) and excellent enantioselectivities (up to 96% ee). Only the diastereomeric ratio was low, which was compensated by easy chromatographic separation. Reactions with *β*-substituted enals gave similar good enantioselectivities and without presenting significant diastereoselectivity issues.

**Scheme 24:**
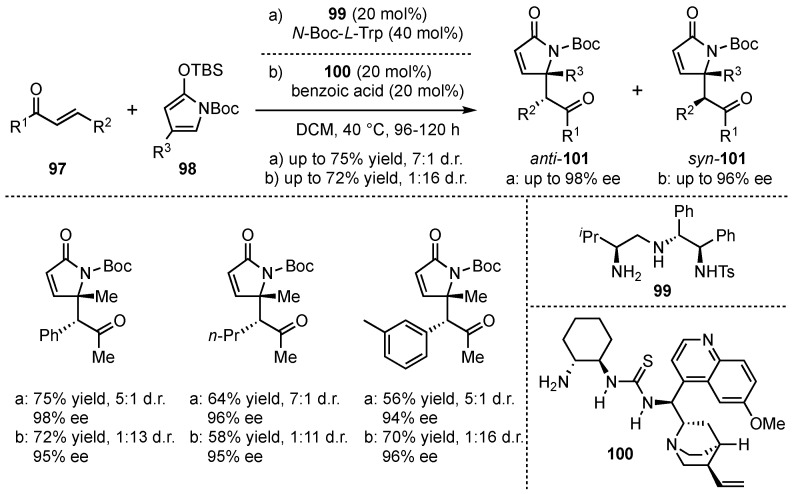
Diastereodivergent method for chiral amine organocatalyzed VMMcR with *N*-Boc-silyloxy-pyrrole-substrates by Ye and Dixon [66].

Simultaneously, Ye, Dixon and coworkers searched for a diastereodivergent method of the VMMcR [66]. They succeeded by screening different multifunctional catalyst structures within the reaction of *γ*-substituted-*N*-Boc-silyloxy-pyrroles **98** with *α*,*β*-unsaturated linear enones **97** (Scheme 24). The primary-amine catalyst **99** facilitated the reaction toward the corresponding *anti*-**101** (up to 7:1 *anti*:*syn*), while the use of quinine-substituted thiourea **100** led to the *syn*-derivative **101** as major product (up to 16:1). Furthermore, the reactions proceed with moderate to good yields (up to 75%) and excellent enantioselectivities (up to 99% ee). 

**Scheme 25:**
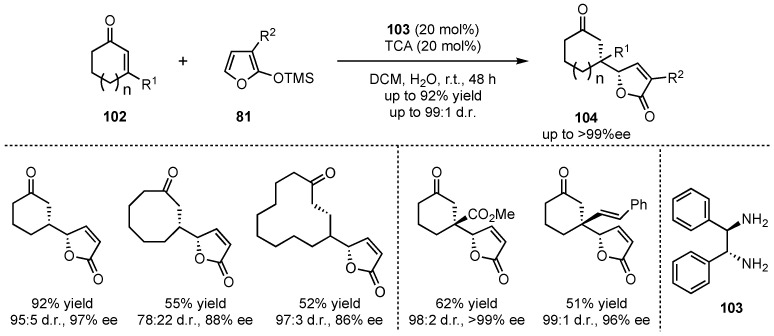
Amplification of the chiral amine catalyzed VMMcR toward cyclic enone-substrates investigated by Singh et al. [67].

Shortly after, the group of Singh sought for a method that also tolerates the use of cyclic enones [67]. In that matter, the chiral 1,2-diphenylethane-1,2-diamine (**103**) efficiently catalyzed the reactions between 2-silyloxyfurans **81** and selected cyclic enones **102** with different ring-sizes (5, 8, 12, and 15 carbons), leading to high enantio- and diasteroselectivities (up to 97% ee and 97:3 d.r.) (Scheme 25). Interestingly, the reactions with 3-substituted cyclic enones, which led to the formation of quaternary carbon-centers in *β*-position, exhibited exceptional selectivities (up to >99% ee and 99:1 d.r.) in the respective products **104**. 

**Scheme 26:**
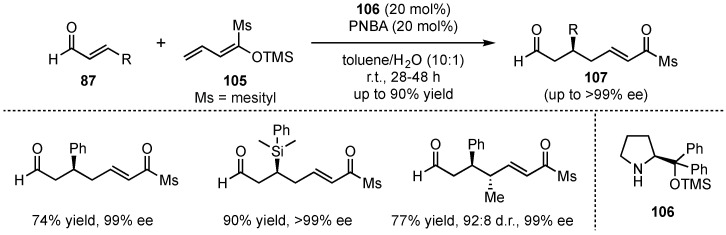
Highly regio- and enantioselective organocatalyzed VMMcR pioneering the employment of acyclic silyl-dienolates presented by Schneider et al. [68].

In 2012, Schneider et al. presented the first approach of a VMMcR with acyclic silyl-dienolates and acyclic *α*,*β*-unsaturated carbonyl-compounds (Scheme 26) [68]. This method bears high challenges in terms of regioselectivity considering the 1,2- and 1,4-reactivity of the applied electrophiles, as well as the *α*- and *γ*-reactivity of the dienolates. Therefore, four different regioisomers might be generated, highlighting the need for precise stereocontrol. Although all Michael reactions enable these isomers, earlier publications circumvent this issue by applying cyclic reaction partners, which have higher tendencies to form the desired 1,7-dioxo-compounds (*γ*-1,4-reactivity). However, in this approach, Schneider et al. were able to overcome the regioselectivity problems by applying the Jørgensen–Hayashi amine catalyst (**104**) to VMMcRs between *α*,*β*-unsaturated aldehydes **87** and linear silyl dienol ethers **105**. After optimization of the process, only the desired 1,7-dioxo products were obtained. It was observed that sterically demanding dienolates provided the best selectivities due to their hindered *α*-reactivity. Follow-up reactions with different substrates exhibited that the desired 1,7-dioxo products **107** could be received in very good yields (up to 90%) and exceptional enantioselectivities (up to >99% ee) for aromatic aldehydes. However, aliphatic substrates gave diminished results. Interestingly, a study on *γ*-methyl-dienolates revealed that their *E*/*Z*-configuration has a direct influence on the diastereoselectivity of the reaction. Hence, the *Z*-dienolate provided *anti*-**107** as the major isomer, while the *E*-analog gave rise to the *syn*-**107** in higher ratios.

**Scheme 27:**
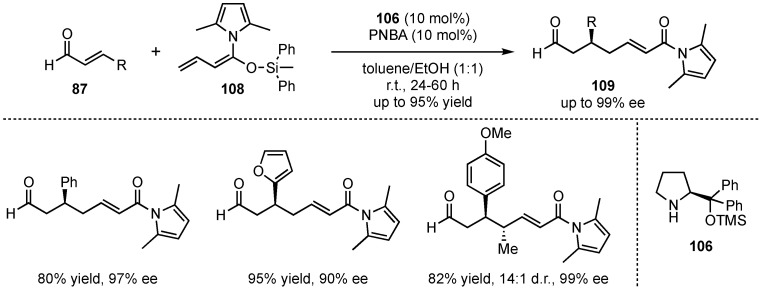
Improvement toward lower catalyst loadings and simpler product derivatization concerning the organocatalyzed VMMcR with acyclic dienolates.

In a later publication, the same group sought for a modification of the process that allows for lower catalyst loadings, as well as for later derivatization of the final products [69]. To combine both requests, they employed vinylketene silyl *N*,*O*-acetals **108** as nucleophiles (Scheme 27). These possess higher reactivities, enabling lower catalyst loadings. Moreover, the formed *N*-acyl pyrrole-products **109** can be more easily converted into relevant motives. After a re-optimization of the reaction conditions and subsequent substrate screening of aldehydes **87**, the same tendencies were observed, in which excellent yields (up to 95%) and enantioselectivities (up to 99% ee) were received for (hetero)aromatic and *β*-silyl-substituted aldehydes. However, aliphatic enals exhibited no reactivity under these conditions. Finally, next to reducing the catalyst loading from 20 mol% to 10 mol%, target derivatizations were also accomplished successfully in follow-up reactions such as hydrolysis, transesterifications, reductions, or alkylations.

**Scheme 28:**
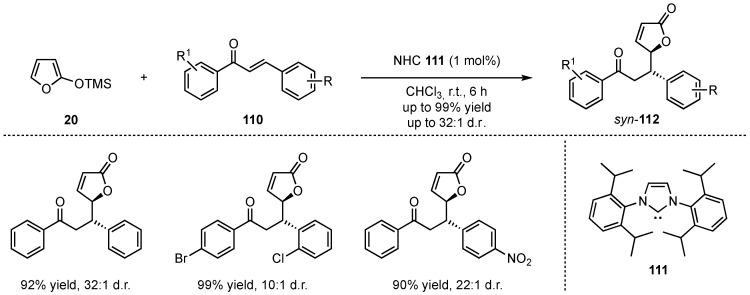
NHC-organocatalyzed diastereoselective vinylogous Mukaiyama Michael reaction presented by Huang, Dai, and He [70].

In 2015, Huang, Dai, and He developed the first application of a *N*-heterocyclic carbene (NHC)-catalyzed VMMcR featuring the addition of 2-(trimethylsilyloxy)furan (**20**) to different chalcone derivatives **110** (Scheme 28) [70]. A mechanism was proposed in which an activated hypervalent silicate nucleophile is formed by attack of the NHC **111** to the silicon atom within the silyl-dienolate **20**. Consequently, the desired products **112** were obtained in high yields (up to 99%) and excellent diastereoselectivities (up to 32:1). Although this method was not enantioselective, the excellent results indicate a high potential for future investigation within chiral NHC catalysts.

## 5. Conclusions

This review provides an overview of the effective organocatalytic approaches within asymmetric vinylogous versions of very common C–C bond formation reactions with silyl-protected dienolates. More specifically, the areas of asymmetric vinylogous Mukaiyama aldol (VMAR), Mannich (VMMnR), and Michael reactions (VMMcR) are presented. Although the organocatalytic methodologies were only developed within the last 20 years, most published studies already present excellent results, especially with regard to the enantiocontrol.

The vast number of organocatalytic structures and their simple tunability allows to tailor these catalysts precisely to the corresponding targeted process. Thus, it was discovered that VMARs are best facilitated by H-bond donor catalysts (e.g., TADDOLS, thioureas, and squaramides), while VMMnRs provide the best results in the presence of chiral Brønsted acids (e.g., disulfonimides, BINOL- or VAPOL-based phosphoric acids). The latter additionally exhibits the first application of anion-binding catalysis in this field. While the above-mentioned reaction types are activated by non-covalent interactions, VMMcRs are found to be catalyzed most efficiently by covalent bonding with primary and secondary amines (e.g., MacMillan-type or Jørgensen–Hayashi catalysts).

As a consequence of the different reaction sites within the nucleophiles (*α*- and *γ*-reactivity), the investigation of vinylogous reactions commonly provokes regioselectivity issues. However, the organocatalytic approaches discussed in this review mostly accomplished the formation of pure *γ*-products, which is admittedly often controlled by the application of cyclic silyl-dienolates. Remarkably, in the past few years, it has been possible to develop reactions with intrinsically less selective acyclic dienolates in a VMMcR with the exclusive formation of *γ*-1,4-adducts.

However, there are still some important limitations within this field that need to be resolved. For instance, the commonly used large catalyst loadings (often 10–20 mol%) need to be reduced, since they complicate potential future applications in industrial processes. Nonetheless, some studies show that this obstacle can be overcome, thus underlining the capability of these organocatalyzed vinylogous C–C-bond formations in the presence of silyl-protected dienolates. Lastly, better and general control with acyclic silyl-dienolates remains highly challenging as well as achieving high levels of stereoselectivity with certain types of aliphatic substrates.

## Data Availability

Not applicable.
